# Exploring Healthcare Professionals’ Practices and Attitudes towards Monitoring and Reporting of Severe Adverse Drug Reactions

**DOI:** 10.3390/healthcare10061077

**Published:** 2022-06-10

**Authors:** Warisara Srisuriyachanchai, Anthony R. Cox, Narumol Jarernsiripornkul

**Affiliations:** 1 Division of Clinical Pharmacy, Faculty of Pharmaceutical Sciences, Khon Kaen University, Khon Kaen 40002, Thailand; warisara.sr@kkumail.com; 2 School of Pharmacy, Institute of Clinical Sciences, College of Medical and Dental Sciences, University of Birmingham, Birmingham B15 2TT, UK; a.r.cox@bham.ac.uk

**Keywords:** practice, attitude, severe adverse drug reaction, monitoring, healthcare professionals

## Abstract

Healthcare professionals (HCPs) play a key role in the monitoring of severe adverse drug reactions (ADRs). The present study aims to explore practices and barriers of HCPs in severe ADR monitoring and reporting, to evaluate their attitudes towards the monitoring and to assess the related factors. Self-administered questionnaires produced in hard copy and Google form were sent to 510 HCPs by stratified random sampling. Of the 350 HCPs that responded (68.6%), 44.9% had ever monitored ADRs. The most common practices were the observation of abnormal symptoms for ADR identification (88.5%), discontinuation of the suspected drug for ADR management (88.5%) and advice on recurrent drug allergy for ADR prevention (88.5%). Most HCPs (93.0%) obtained further patient history to identify severe ADRs. The uncertainty of the causal relationship was a major barrier to ADR reporting (60.0%). Pharmacists were more involved with practices in ADR monitoring and reporting (OR 20.405; *p* < 0.001), whereas longer work experience (>20 years) was negatively related to the practices (OR 0.271; *p* = 0.024). Over one-third (37.6%) of HCPs had a positive attitude towards severe ADR monitoring. In conclusion, the practices in severe ADR monitoring varied among different professions. However, the barriers to the reporting of ADRs still exist; hence, improving knowledge and cooperation among HCPs should be promoted.

## 1. Introduction

Pharmacovigilance is a key part of monitoring ADRs around the world. The detection, assessment, management, prevention and reporting of suspected ADRs is the responsibility of HCPs in adverse drug reaction monitoring systems [1]. Physicians, pharmacists and nurses make up the majority of HCPs in Thailand and are the primary resource for reporting severe ADRs.

In Thailand, physicians and nurses are mainly responsible for direct patient care with pharmacists providing medication and dispensing and counseling services, during which pharmacists obtain additional information from the patients that could identify potential ADRs. Therefore, pharmacists play an important role in ADR monitoring systems and reporting ADRs to the Health Product Vigilance Center (HPVC) in Thailand. The spontaneous reporting system (SRS) that utilizes HCP reporting of ADRs is, globally, the most commonly employed method for pharmacovigilance. Such systems are a rapid and effective way to collate suspected ADRs, but they have a well-known limitation of under-reporting. One comprehensive systematic review found that only 6% of potential ADRs are reported in spontaneous reporting systems [2]. The success or failure of any SRS depends on the intrinsic and extrinsic factors [3]. The intrinsic factors are the knowledge, skill and attitudes of the reporters, while the extrinsic factors are related to the systems employed to identify and report ADRs [4,5,6]. The involvement of all of the HCPs that provide patient care in the ADR reporting process is one key to good post-marketing safety surveillance [7], but, in recent years, the increased involvement of patients in the ADR reporting process in many countries has led to an increased reporting of ADRs [8,9,10,11]. Differences exist in the types of ADRs reported by direct patient reports and by HCPs. HCPs report a higher proportion of serious ADRs that result in death, hospitalization and prolongation of hospital stay than patients [10]. This is because HCPs are more likely to become involved with patients when they experience more severe ADR.

While most studies show that physicians, pharmacists and nurses have a reasonable knowledge of, and attitudes towards, ADR reporting [12,13,14,15,16,17,18,19,20,21,22,23], the under-reporting of severe ADRs remains a serious concern. The aims of this study are to identify the practices methods concerning severe ADR monitoring, to explore the barriers to ADR reporting, the factors affecting practices in ADR monitoring and reporting, and attitudes towards severe ADR monitoring among physicians, pharmacists and nurses in Thailand. 

## 2. Materials and Methods

This research is a cross-sectional study conducted at two university hospitals in northeast Thailand from June to September 2020. Eligible participants were physicians, pharmacists and nurses who worked at clinical departments in the two hospitals. The study excluded HCPs without Internet access. The sample-size calculation for the study was determined using Taro Yamane [24]. The minimum appropriate number of participants was 350, assuming a participation refusal rate of 45.2% from a previous study [25]. A total sample size of 510 enrolled participants was chosen, consisting of 134 physicians, 69 pharmacists and 307 nurses by stratified sampling.

The questionnaire for self-administration was developed by the research team following previous relevant studies [12,17,26,27]. Three HCPs with expertise in the field of pharmacovigilance and ADRs (one physician, one clinical pharmacist and one nurse) evaluated the developed questionnaire for content validity. The index of consistency (IOC) was calculated to assess the internal consistency [28,29,30]. All the questions in the draft questionnaire passed content validity testing with an IOC > 0.5 for each item. The questionnaire was adjusted and piloted using 15 HCPs from other hospitals. The 15 HCPs were asked to complete the questionnaire and they were then asked to comment on each question individually, in terms of ease of understanding and for any general recommendations for improving the questionnaire. The questionnaire was appropriately re-adjusted following the suggestions from the pilot study, and subjected to validation testing to obtain the final version. HCPs were asked about all ADRs from any types of drugs that the HCPs had ever monitored in all departments of the hospitals. The final questionnaire consisted of the three following sections:

Section 1: Closed questions were used with a checklist to obtain demographic data on gender, education level, profession, income, inpatient or outpatient department, number of patients per day and time spent per patient. Open questions were used to obtain data on age and time since professional qualification.

Section 2: Closed questions were used with checklists to explore the severe ADR monitoring methods and barriers towards ADR reporting experienced by HCPs. 

Section 3: Attitudes towards severe ADR monitoring methods. This section contained 8 questions made up of 4 positive statements (1, 4, 7 and 8) and 4 negative statements (2, 3, 5 and 6). The agreement with each statement was measured on a 5-point Likert scale. The scores ranged from 1 to 5 for strongly disagree to strongly agree for positive statements and from 1 to 5 for strongly agree to strongly disagree for negative statements. The total score was calculated by summing the scores for all questions for a total score range from 8 to 40. The total score range was divided into three equal parts, categorized as poor (8–18), moderate (19–29) and good (30–40) attitudes.

The final questionnaire was constructed as both a hard copy and as Google Documents form that could be accessed via a QR code or a URL (both provided in the letter of invitation). The online version of the self-administered questionnaire was also tested in the pilot study. Letters of invitation containing a hard copy of the questionnaire were directly distributed to the HCPs through the various Heads of Department by stratified sampling. After 2 weeks, non-responders were sent a reminder letter with a hard copy of the questionnaire. Two weeks later, non-responders were sent a second reminder letter and another hard copy of the questionnaire. The final collection of the responses was completed at sixteen weeks from the date of distribution of the first questionnaire. Questionnaires received after this date were excluded from the analysis. The survey was conducted from June to September 2020.

Questionnaire responses were entered into SPSS for Windows version 26.0 for analysis. Demographic data, frequency, types of severe ADR monitoring method and attitude scores were analyzed using descriptive statistics. Pearson’s chi-squared and Fisher’s exact test were used to compare subgroups for categorical data, and independent-sample *t*-tests or ANOVA were used for continuous variables with normal distributions. The Mann–Whitney U or Kruskal–Wallis tests were used to determine the distribution for the continuous data of non-parametric variables. Univariate analysis of the factors related to the variables was analyzed using the chi-squared test. The variables associated with the practices of HCPs in ADR monitoring and reporting with *p*-values < 0.25 in the univariate analyses were entered into a multivariate analysis. Multivariate analysis of the factors related to xpractices of HCPs in ADR monitoring and reporting was analyzed using logistic regression. The results with *p*-values less than 0.05 were considered statistically significant. The study was approved by the Ethics Committee of the Khon Kaen University Ethics Committee for Human Research (Number HE621444 on 16 December 2019). 

## 3. Results

### 3.1. Response Rate

A total of 510 questionnaires were distributed by hand to HCPs at the two hospitals. Of the 350 valid questionnaires that were returned (68.6%), 314 were from Srinagarind Hospital (89.7%) and 36 were from the Queen Sirikit Heart Center of the Northeast (10.3%). The 350 respondents comprised 65 physicians (18.6%), 29 pharmacists (8.3%) and 256 nurses (73.1%). 

### 3.2. Demographic Data

The majority of respondents were female (88.9%) aged 18–34 years (59.7%) and had a bachelor’s degree (82.0%). Almost two-thirds of the respondents (60.3%) had less than 10 years’ work experience (Table 1).

### 3.3. Methods of ADR Monitoring

A total of 191 (54.6%) respondents claimed that they knew about ADR monitoring methods in Thailand, and 157 (44.9%) respondents said that they had been involved in ADR monitoring. Among these 157 respondents, the most frequently known ADR identification method was the observation of abnormal symptoms following the administration of drugs (88.5%) (Table 2). The top three frequently used methods of ADR identification were the same, with the observation of abnormal symptoms after the administration of drugs reported by 126 respondents (80.3%), followed by reports from patients (56.7%) and high-alert drug lists (35.7%). For the physicians and nurses, the observation of abnormal symptoms following the administration of drugs was the most well-known ADR identification method (100.0% and 89.8%, respectively). For the pharmacists, a report from the patients was the most common known ADR identification method (85.2%). An awareness of the high-alert drug list ADR identification method was not significantly different between professions (*p =* 0.441), but an awareness of all the other methods of ADR identification were significantly different between professions (*p* < 0.05). The practice of these methods of severe ADR identification varied among the respondents, but the further patient history taking was used by nearly all the respondents (93.0%) with no statistical difference between professions (*p =* 0.271). Other methods were less well-known using specific ADR criteria (18.5%), the next most common among all respondents, but there was a significant difference between professions (*p =* 0.001). There was also a significant difference between professions for drug-gene testing (*p =* 0.003) and confirmation by additional laboratory data (*p* < 0.001) (Table 2). 

Approximately half of the respondents (49.0%) did not know about the causality assessment of ADRs. Of the 157 respondents who had been involved in ADR monitoring, 39.5% and 21.7% had known the WHO-UMC criteria and Naranjo’s algorithm, respectively. The awareness of Naranjo’s algorithm was significantly different between professions (*p* < 0.001) (Table 2), due to the very high rate of awareness among the pharmacists (96.3%). Only 24.8% of the respondents claimed that they used causality assessment methods, with Naranjo’s algorithm (69.2%), and consulted with the HCP team (59.0%) in relation to the most frequently reported methods. 

The method for ADR management most frequently reported by the HCPs was stopping the suspected drug (88.5%). The used methods of ADR management were significantly different between professions, except for providing patient advice about drug use (*p =* 0.497) and monitoring patients (*p =* 0.296) (Table 2). The methods for ADR prevention most frequently reported by the HCPs was providing patient advice about recurrent drug allergies (88.5%). The common ADR prevention methods were different among the professions. There were significant differences between the professions in relation to the awareness of different ADR prevention methods, except for providing patient advice about recurrent drug allergies (*p =* 0.370), transferring drug allergy data to a responsible agency (*p =* 0.221), search data from ADR reference books (*p =* 0.213) and attaching drug allergy labels to the patient’s bed (*p =* 0.908) (Table 2). 

Of the 157 respondents who were involved in ADR monitoring, 81.5% claimed that they reported all suspected ADR cases. Table 2 shows that the respondents most commonly reported suspected ADR symptoms to the responsible physicians (81.5%), followed by the pharmacists on ADR duty (79.0%) and the responsible nurses (60.5%). Only pharmacists reported suspected ADRs to the Ministry of Public Health (18.5% of pharmacist respondents).

### 3.4. Barriers to ADR Reporting

Among 155 respondents, the top three barriers to ADR reporting were the uncertainty of a causal relationship between drug and reactions (60.0%) well-known ADRs (22.6%), not understanding the processes and steps of ADR monitoring and unavailability of ADR reporting forms (both 19.4%). The rates of not understanding the processes and steps of ADR monitoring (*p* = 0.017), inadequate time for ADR reporting (*p* = 0.001) and the shortage of staff (*p* = 0.022) were significantly different between professions (Table 3). 

### 3.5. Factors Related to Practices in ADR Monitoring and Reporting

The univariate analysis of the factors related to practices in ADR monitoring showed that hospital (*p* = 0.015), gender (*p* = 0.027), age (*p* = 0.025), profession (*p* < 0.001) and years of work experience (*p* = 0.030) were significantly associated with practices in the monitoring and reporting ADRs. Multiple logistic regression analysis identified pharmacists’ professions (OR 20.405; 95% CI 4.098, 101.607; *p* < 0.001) and more than 20 years of work experience (OR 0.271; 95% CI 0.087, 0.845; *p* = 0.024) as the factors independently associated with reporting ADRs (Table 4).

### 3.6. Attitudes towards Severe ADR Monitoring

Attitudinal scores could be calculated for 157 respondents. The overall mean attitude score was 28.5 ± 3.27 (min-max = 8–40). Just under under two-thirds of respondents (62.4%) had a moderate attitude (mean = 26.5 ± 2.16); the remaining 59 respondents (37.6%) had a good attitude (mean = 31.8 ± 1.82). Physicians had the highest overall mean attitude score, followed by pharmacists and nurses (29.3 ± 2.53, 28.9 ± 2.32, and 28.2 ± 3.57, respectively), and the overall mean attitude scores were not significantly different between the three professions (*p* = 0.121). 

A large majority of the respondents agreed with the following statements: severe ADRs are manageable and preventable (90.5%), the management of severe ADRs can improve patient compliance (89.1%) and an ADR monitoring tool can decrease the severity level of ADRs (87.3%). Conversely, approximately two-thirds of the respondents disagreed with the statement that the management of severe ADRs is a waste of time (63.0%), half of the respondents disagreed with the statement that the treatment of severe ADRs is the responsibility of HCPs only (49.1%) and one-third of some respondents disagreed with the statement that the monitoring of severe ADRs is difficult and complicated (32.5%) (Table 5).

## 4. Discussion

This cross-sectional study had a reasonable overall response rate of 68.6%, slightly lower than reported in comparable previous studies [17,26], but with a similar majority of female respondents [17]. The proportions of HCPs by profession in our study (physicians 18.6%, pharmacists 8.3% and nurses 73.1%) were broadly similar to those reported in the previous studies (physicians 13.8%, pharmacists 8.08% and nurses 77.4% and physicians 17.6%, pharmacists 20.5% and nurses 61.9%) [17,26]. The key element to identify a suspected ADR in the current study was the temporal relationship between the administration of a medicine and the observation of an adverse effect. Overall, the known general methods of ADR identification in the current study were similar to the previous studies [31,32]. Pharmacists most frequently used reports from patients to identify ADRs; physicians and nurses used the observation of abnormal symptoms after the drugs were administered. This could be because physicians and nurses are more able to directly observe patients’ symptoms. Pharmacists also used ADR monitoring systems by HCP teams to identify ADRs much more frequently than physicians and nurses. Patient history taking was the most common way of identifying severe ADRs, in line with the previous studies [33], and using specific criteria for severe ADR identification was needed, as reported in the previous studies [34,35,36]. This suggests that the selection of methods for ADR identification by HCPs depends on their pattern of patient care.

Few respondents were aware of causality tools, such as the WHO-UMC criteria and Naranjo’s algorithm, despite reports of the widespread use of these tools [37,38]. Therefore, the strategies to increase knowledge about the causality assessment methods of ADRs should be established for all HCPs. Pharmacists were the main profession to use these tools for causality assessments. The most frequently used method for causality assessment by physicians and pharmacists was the Naranjo’s algorithm, although pharmacists pre-dominated in this category. The most frequently selected methods for ADR management by all HCPs were to stop the suspected drugs, provide patient advice about drug use and changing new drugs, which is also in line with a previous study [12]. However, the study was conducted in a primary healthcare center that used referral to a tertiary health facility as ADR management. This result differs from our study conducted in tertiary care teaching hospitals, where referral was not used. The prevention of the suspected drug was suitable for severe ADR management. We found that physicians and pharmacists were, in general, far more involved in ADR management than nurses.

This study found that the most used methods of ADR prevention by all professions were providing patient advice about recurrent drug allergy and recording ADR history in medical notes, which is also in line with the previous studies [26,39]. This indicated that HCPs were aware of patient safety, especially concerning the information about recurrent drug allergies in patients. The individual professions used different methods of ADR prevention, which seemed to be aligned to their professional role. The physicians mainly focused on the methods that directly affected the patients and recorded safety data in medical notes rather than using systemic processes, such as recording the ADR history in computer database or attaching drug allergy stickers. The pharmacists usually started with drug allergy card provision to patients, followed by advice to patients, recording the ADR history and attaching allergy labels to the medical notes. The evidence from the previous studies suggests that hospital pharmacists can not only identify and report ADRs, but also help in the prevention of ADRs [40], and this is one of the main roles of pharmacists in Thailand [39]. It can be observed from our survey that the most frequently employed method of ADR prevention by nurses was transferring drug allergy data to a responsible agency or hospital management, presumably because of their close patient contact in nursing care. Responsible physicians were the most likely to receive ADR reports from other HCPs. This finding was comparable to other studies [41,42,43,44,45]. The nurses usually reported ADRs to the responsible physicians and pharmacists who were on ADR duty, rather than directly to the pharmacy department in the hospitals, as can be observed in the previous studies [41,45]. The pharmacists had the highest proportion of reporting ADRs to the regulatory authority. In Thailand, pharmacists have the responsibility of reporting ADRs to the Thai Health Product Vigilance Center, and our study confirmed that pharmacists understand this role.

The most common barrier to ADR reporting by all professions was the uncertainty about the causal relationship between drug and reactions. Similar results were found in the previous studies of pharmacists [19,23,27,46,47,48] and nurses [42]. This is regrettable, since regulatory authorities only require a suspicion that a drug was linked to an adverse effect. The knowledge of a causal relationship should be promoted among HCPs. The other barriers to ADR reporting found in this study were at rates similar to those found in the previous studies [4,12,16,19,23,42,44,46,47,48,49,50]. However, in our study, not understanding the ADR monitoring process was a frequent barrier to ADR reporting by physicians, in contrast to the previous studies that showed that the main barrier was unavailable ADR reporting forms [12,16,51]. Not only improving the knowledge of causal relationships, but also understanding the ADR monitoring process should be promoted. For nurses, the main barrier to ADR reporting was the unavailability of ADR reporting forms, which matched similar results obtained in the previous studies [4,44,50]. Therefore, providing adequate ADR reporting forms will support nurses to increase ADR reporting. However, in our study, it was only nurses that had a fear of legal complaints as a barrier to ADR reporting, in contrast to the previous studies that showed that physicians, pharmacists and nurses all had a fear of the legal issues [12,13,48]. The profession of the HCPs and the years of work experience were significantly associated with the practices in ADR monitoring and reporting. The pharmacists were more likely to monitor and report ADRs, which is in line with the pharmacists having the responsibility of monitoring and reporting ADRs to the national pharmacovigilance system in Thailand. A recent study found that senior pharmacists were more likely to report ADRs than general pharmacists [49], and that physicians with more than six years of work experience were 4.6 times more likely to report an ADR, compared to physicians with one to three years of work experience [52]. Our study shows that pharmacists in practice for less than 10 years are more likely to monitor ADRs than pharmacists practicing for more than 20 years, which is in agreement with a previous study [53]. Other studies also reported that having more than 10 years of work experience was associated with poor ADR reporting practice by HCPs [26], and that younger pharmacists and those who had received ADR training were significantly more likely to report ADRs [48].

The majority of respondents had a positive attitude towards ADR monitoring or reporting. Our study shows that around 40% of all HCPs have a positive attitude towards severe ADR monitoring, which is a lower proportion than the previous studies [12,17,26,27]. However, our study includes all the steps in the monitoring and reporting of severe ADRs, whereas the other studies only measure the attitudes towards ADR reporting. Our HCPs agreed that the management of severe ADRs could improve patient compliance, confirming a previous study that found that ADRs influence medication adherence [54]. In the current study, the HCP respondents agreed that it can be difficult to differentiate between severe ADRs and adverse events with other causes, as found in the previous studies [42,46]. 

The current study has some limitations. It was conducted only in the northeastern region of Thailand; hence, our findings may not be generalized to all HCPs in Thailand. Moreover, the gathered findings were obtained from self-administered questionnaire, which may be subject to recall and social desirability biases.

## 5. Conclusions

HCPs frequently used the further patient history taking as the main method of severe ADR identification. The uncertainty of the causal relationship between drugs and reactions was a major barrier to reporting ADRs. HCPs with less work experience and with a pharmacist profession were more likely to monitor and report ADRs. However, HCPs had a positive attitude towards severe ADR monitoring. Improving the knowledge of ADR monitoring should be promoted to all HCPs. This could enhance the awarness of HCPs to recognize the importance of ADR monitoring and reporting, which would lead to medication safety for patients.

## Figures and Tables

**Table 1:** Respondent demographic characteristics.

Characteristic	Profession of Respondents, N (%)			
	Physician (*n* = 65)	Pharmacist (*n* = 29)	Nurse (*n* = 256)	Total (*n* = 350)
Hospital				
Srinagarind Hospital	63 (96.9)	21 (72.4)	230 (89.8)	314 (89.7)
Queen Sirikit Heart Center	2 (3.1)	8 (27.6)	26 (10.2)	36 (10.3)
Gender				
Male	32 (49.2)	1 (3.4)	6 (2.3)	39 (11.1)
Female	33 (50.8)	28 (96.6)	250 (97.7)	311 (88.9)
Age (years)				
18–34	48 (73.8)	11 (37.9)	150 (58.6)	209 (59.7)
35–50	15 (23.1)	17 (58.6)	70 (27.3)	102 (29.1)
>50	1 (1.5)	1 (3.4)	36 (14.1)	38 (10.9)
Mean ± S.D.	29.8 ± 6.51	37.7 ± 7.50	36.2 ± 10.52	35.2 ± 10.00
Median (range)	27 (23–53)	39 (27–57)	33 (21–66)	32 (21–66)
Routine work				
OPD	56 (96.2)	28 (96.6)	82 (32.0)	166 (47.4)
IPD	60 (92.3)	23 (79.3)	211 (82.4)	294 (84.0)
Both	51 (78.5)	22 (75.9)	37 (14.5)	110 (31.4)
Highest education level				
Bachelor’s degree	39 (60.0)	14 (48.3)	234 (91.4)	287 (82.0)
Master’s degree or higher	26 (40.0)	15 (51.7)	22 (8.6)	63 (18.0)
Years of work experience (years)				
<10	56 (86.2)	13 (44.8)	142 (55.5)	211 (60.3)
10–20	8 (12.3)	11 (37.9)	50 (19.5)	69 (19.7)
>20	1 (1.5)	5 (17.2)	64 (25.0)	70 (20.0)
No. of patients per day (cases)				
<10	8 (12.3)	1 (3.4)	105 (41.0)	114 (32.6)
10–30	46 (70.8)	0 (0.0)	107 (41.8)	153 (43.7)
>30	11 (16.9)	28 (96.6)	44 (17.2)	83 (23.7)
Time spent on care per patient (min)				
<20	43 (66.2)	27 (96.4)	56 (21.9)	126 (36.1)
>20	22 (33.8)	1 (3.6)	200 (78.1)	223 (63.9)
Proportion of time spent in direct patient contact				
<50% of all working time	34 (52.3)	11 (37.9)	76 (29.7)	121 (34.6)
>50% of all working time	31 (47.7)	18 (62.1)	180 (70.3)	229 (65.4)
No. of ADRs identified in the previous year				
<20	18 (27.7)	5 (17.2)	96 (37.5)	119 (34.0)
>20	4 (6.2)	21 (72.4)	12 (4.7)	37 (10.6)

S.D.: standard deviation; OPD: outpatient department; IPD: inpatient department; No.: number.

**Table 2:** Methods of ADR monitoring by profession.

Method	Profession of Respondents, N (%)				*p*-Value ^a^
	Physician (*n* = 22)	Pharmacist (*n* = 27)	Nurse (*n* = 108)	Total (*n* = 157)	
**General ADR identification methods**					
Observe abnormal symptoms	22 (100.0)	20 (74.1)	97 (89.8)	139 (88.5)	0.015 ^b,^*
High-alert drug list	11 (50.0)	12 (44.4)	62 (57.4)	85 (54.1)	0.441
Abnormal laboratory data	11 (50.0)	13 (48.1)	18 (16.7)	42 (26.8)	<0.001 *
Alerting orders	10 (45.5)	14 (51.9)	24 (22.2)	48 (30.6)	0.003 *
Trigger tools or antidotes	8 (36.4)	18 (66.7)	24 (22.2)	50 (31.8)	<0.001 *
Report from patients	13 (59.1)	23 (85.2)	58 (53.7)	94 (59.9)	0.012 *
HCP team ADR monitoring systems	10 (45.5)	19 (70.4)	32 (29.6)	61 (38.9)	<0.001 *
**Additional methods for identification of severe ADRs**					
Drug-gene testing	5 (22.7)	8 (29.6)	8 (7.4)	21 (13.4)	0.003 ^b,^*
Skin test	3 (13.6)	6 (22.2)	12 (11.1)	21 (13.4)	0.279 ^b^
Additional patient history taking	20 (90.9)	27 (100.0)	99 (91.7)	146 (93.0)	0.271 ^b^
Additional laboratory data	5 (22.7)	8 (29.6)	3 (2.8)	16 (10.2)	<0.001 ^b,^*
Use specific ADR criteria ^c^	4 (18.2)	12 (44.4)	13 (12.0)	29 (18.5)	0.001 ^b,^*
**Recognize methods of ADR causality assessment**					
WHO-UMC criteria	8 (36.4)	14 (51.9)	40 (37.0)	62 (39.5)	0.352
Naranjo’s algorithm	8 (36.4)	26 (96.3)	0 (0.0)	34 (21.7)	<0.001 *
**ADR management methods**					
Stop the suspected drug	22 (100.0)	27 (100.0)	90 (83.3)	139 (88.5)	0.005 ^b,^*
Change to alternative drug	17 (77.3)	19 (70.4)	20 (18.5)	56 (35.7)	<0.001 *
Use additional drug to treat ADR symptoms	10 (45.5)	14 (51.9)	1 (0.9)	25 (15.9)	<0.001 ^b,^*
Decrease drug dose	6 (27.3)	9 (33.3)	6 (5.6)	21 (13.4)	<0.001 ^b,^*
Change drug administration time	4 (18.2)	4 (14.8)	4 (3.7)	12 (7.6)	0.013 ^b,^*
Change drug administration rate	5 (22.7)	15 (55.6)	8 (7.4)	28 (17.8)	<0.001 ^b,^*
Change drug dosage form	7 (31.8)	1 (3.7)	2 (1.9)	10 (6.4)	<0.001 ^b,^*
Advise patients about the drug	11 (50.0)	18 (66.7)	63 (58.3)	92 (58.6)	0.497
Monitor patient	5 (22.7)	8 (29.6)	18 (16.7)	31 (19.7)	0.296
**ADR prevention methods**					
Advise patients about recurrent drug allergy	20 (90.9)	26 (96.3)	93 (86.1)	139 (88.5)	0.370 ^b^
Drug allergy card	15 (68.2)	27 (100.0)	41 (38.0)	83 (52.9)	<0.001 *
Transfer drug allergy data to responsible agency	13 (59.1)	18 (66.7)	82 (75.9)	113 (72.0)	0.221
Adjust drug dose in special populations	10 (45.5)	9 (33.3)	9 (8.3)	28 (17.8)	<0.001 ^b,^*
Check drug interactions	12 (54.5)	11 (40.7)	23 (21.3)	46 (29.3)	0.003 *
Search ADR reference books	4 (18.2)	8 (29.6)	16 (14.8)	28 (17.8)	0.213 ^b^
Record ADR history in medical notes	16 (72.7)	24 (88.9)	44 (40.7)	84 (53.5)	<0.001 *
Record ADR history in computer programs	12 (54.5)	25 (92.6)	37 (34.3)	74 (47.1)	<0.001 *
Attach drug allergy sticker to medical notes	6 (27.3)	25 (92.6)	43 (39.8)	74 (47.1)	<0.001 *
Attach drug allergy label to the patient’s bed	5 (22.7)	5 (18.5)	24 (22.2)	34 (21.7)	0.908
**Staff**/**organization to whom HCPs reported the ADRs**					
Responsible physicians	12 (54.5)	19 (70.4)	97 (89.8)	128 (81.5)	<0.001 ^b,^*
Pharmacists on ADR duty	21 (95.5)	21 (77.8)	82 (75.9)	124 (79.0)	0.121
Responsible nurses	13 (59.1)	15 (55.6)	67 (62.0)	95 (60.5)	0.818
Pharmacy department	7 (31.8)	13 (48.1)	29 (26.9)	49 (31.2)	0.102
The Ministry of Public Health (MOPH)	0 (0.0)	5 (18.5)	0 (0.0)	5 (3.2)	<0.001 ^b,^*

^a^ Pearson’s chi-squared Test; ^b^ Fisher’s exact test; ^c^ Specific ADR criteria: vancomycin evaluation criteria (*n =* 1), anaphylaxis evaluation criteria (*n =* 1), drug-use manual for hospital (*n =* 10), RegiSCAR (Registry of Severe Cutaneous Adverse Reactions) score for DRESS (*n =* 4), not identified (*n =* 13); * the level of significant different < 0.05.

**Table 3:** Barriers to ADR reporting experienced by profession.

Reasons	Profession of Respondents, N (%)				*p*-Value ^a^
	Physician (*n* = 22)	Pharmacist (*n* = 27)	Nurse (*n* = 106)	Total (*n* = 155)	
Well-known ADRs	6 (27.3)	6 (22.2)	23 (21.7)	35 (22.6)	0.849
Not serious ADRs	5 (22.7)	8 (29.6)	16 (15.1)	29 (18.7)	0.196
Uncertainty of the causal relationship between drug and reactions	17 (77.3)	18 (66.7)	58 (54.7)	93 (60.0)	0.107
Not understanding the ADR monitoring process	9 (40.9)	3 (11.1)	18 (17.0)	30 (19.4)	0.017 *
ADR reporting forms unnavailable	4 (18.2)	2 (7.4)	24 (22.6)	30 (19.4)	0.200
ADR reporting forms too complicated	3 (13.6)	4 (14.8)	5 (4.7)	12 (7.7)	0.078 ^b^
Inadequate time for ADR reporting	6 (27.3)	11 (40.7)	11 (10.4)	28 (18.1)	0.001 ^b,^*
Lack of cooperation between healthcare teams	2 (9.1)	3 (11.1)	7 (6.6)	12 (7.7)	0.581 ^b^
Staff shortage	3 (13.6)	7 (25.9)	8 (7.5)	18 (11.6)	0.022 ^b,^*
Lack of support from leaders	2 (9.1)	0 (0.0)	4 (3.8)	6 (3.9)	0.195 ^b^
Lack of technology to monitor ADRs	4 (18.2)	1 (3.7)	5 (4.7)	10 (6.5)	0.055 ^b^

^a^ Pearson’s chi-squared test; ^b^ Fisher’s exact test; * the level of significant different < 0.05.

**Table 4:** Multiple logistic regression analysis of the factors related to practices in ADR monitoring and reporting.

Variables	No. of Respondents; N (%)		Adjusted OR	95% CI		*p*-Value
	Monitor and Report ADRs (*n* = 157)	Not Monitor and Report ADRs (*n* = 193)		Lower	Upper	
Hospital						
Srinagarind Hospital	134 (85.4)	180 (93.3)	1			0.396
Queen Sirikit Heart Center	23 (14.6)	13 (6.7)	1.418	0.633	3.174	
Gender						
Male	11 (7.0)	28 (14.5)	1			0.152
Female	146 (93.0)	165 (85.5)	1.944	0.783	4.824	
Age (years)						
18–34	91 (58.0)	118 (61.1)	1			
35–50	55 (35.0)	47 (24.4)	2.145	0.929	4.954	0.074
>50	11(7.0)	27 (14.0)	1.728	0.470	6.351	0.410
Profession						
Physician	22 (14.0)	43 (22.3)	1			
Pharmacist	27 (17.2)	2 (1.0)	20.405	4.098	101.607	<0.001 *
Nurse	108 (68.8)	148 (76.7)	1.289	0.626	2.656	0.491
Years of work experience (years)						
<10	96 (61.1)	115 (59.6)	1			
10–20	38 (24.2)	31 (16.1)	0.643	0.276	1.498	0.306
>20	23 (14.6)	47 (24.4)	0.271	0.087	0.845	0.024 *

Variables included in the multiple logistic regression analysis are hospital, gender, age, profession and years of work experience. * The level of significant different < 0.05

**Table 5:** Attitudes of respondents towards severe ADR monitoring.

Statements	Attitudes (N, %)					Mean ± S.D.
	Absolutely Agree	Agree	Not Sure	Disagree	Absolutely Disagree	
Severe ADR monitoring is a direct role of HCPs.	48 (30.6)	85 (54.1)	13 (8.3)	6 (3.8)	5 (3.2)	4.05 ± 0.911
2. Treatment for severe ADRs is the responsibility of HCPs.	11 (7.0)	49 (31.2)	20 (12.7)	64 (40.8)	13 (8.3)	3.12 ± 1.151
3. Monitoring severe ADRs is difficult and complicated.	10 (6.4)	63 (93.6)	36 (53.5)	45 (30.6)	3 (1.9)	2.80 ± 0.992
4. Severe ADRs are manageable and preventable.	29 (18.5)	113 (72.0)	11 (7.0)	1 (0.6)	3 (1.9)	4.04 ± 0.673
5. It can be difficult to differentiate between severe ADRs and symptoms due to other causes.	9 (5.7)	69 (43.9)	48 (30.6)	28 (17.8)	3 (1.9)	2.66 ± 0.903
6. Severe ADR management is a waste of time.	4 (2.5)	27 (17.2)	27 (17.2)	69 (43.9)	30 (19.1)	3.60 ± 1.061
7. Severe ADR management can improve patient compliance.	36 (22.9)	104 (66.2)	17 (10.8)	0 (0.0)	0 (0.0)	4.12 ± 0.570
8. ADR monitoring tools can decrease the severity level of ADRs.	37 (23.6)	100 (63.7)	18 (11.5)	0 (0.0)	2 (1.3)	4.08 ± 0.679

S.D.: standard deviation; N: number of respondents.

## Data Availability

Not applicable.
